# Stir-baked *Xanthii fructus* ameliorates adjuvant arthritis by regulating gut microbiota, short-chain fatty acids and metabolites

**DOI:** 10.3389/fmicb.2025.1599529

**Published:** 2025-06-05

**Authors:** Xinyuan Cui, Zhaoqi Ding, Yujie Ji, Jiale Liu, Zenghui Chang, Junshuo Zhang, Xinyi Wang, Kai Liu, Yuanyuan Liu

**Affiliations:** State Key Laboratory of Advanced Drug Delivery and Release Systems, College of Pharmacy and Institute of Materia Medica, Shandong First Medical University and Shandong Academy of Medical Sciences, Jinan, Shandong, China

**Keywords:** stir-baked *Xanthii fructus*, Rheumatoid arthritis, gut microbiota, metabolomics, short-chain fatty acids

## Abstract

**Introduction:**

Rheumatoid arthritis (RA) is a common and widespread autoimmune disease whose incidence is increasing. Stir-baked Xanthii fructus (XF) is used to treat RA in clinic. However, it’s *in vivo* efficacy and mechanistic pathways remain unclear. This study aimed to explored XF’s therapeutic effects and its mechanisms by comprehensive serum metabolomics and gut microbiota analysis.

**Methods:**

The components in XF were identified using the UPLC-MS technique. A rat model of adjuvant arthritis was established using complete Freund’s adjuvant to evaluate the efficacy of XF. The *in vivo* mechanisms were explored through microbiome, short-chain fatty acid (SCFAs), and metabolomics analysis.

**Results:**

In total, 27 components were identified in XF. The treatment effectively suppressed inflammatory factors and alleviated pannus and cartilage damage. In addition, this article revealed a substantial remodeling of the gut microbiota composition, characterized by a reduced abundance of pro-inflammatory bacteria, increased populations of immunomodulatory bacteria and restored levels of SCFAs. Serum metabolomic profiling identified 17 arthritis-associated metabolites, primarily involved in glycerophospholipid metabolism and bile acid biosynthesis. Then, a strong correlation was found between gut microbiota and serum metabolites, indicating that XF exerts its therapeutic effects through immunomodulation, energy homeostasis regulation, and redox balance maintenance via the gut-joint axis.

**Discussion:**

This study provides new insights for further research into the targeted therapy of XF to ameliorate adjuvant arthritis.

## Introduction

Rheumatoid arthritis (RA) is a systemic autoimmune disease caused by multiple factors. The condition is marked by joint damage, symmetric synovial inflammation and pannus, which can generate joint deformity or loss of joint function. At present, the treatment of RA mostly consists of non-steroidal anti-inflammatory drugs (NSAIDs), disease-modifying antirheumatic drugs (DMARDs), glucocorticoid drugs, and biotechnological drugs (Shah et al., 2022). However, the limited availability of clinically effective RA treatments underscores the urgent need for developing novel therapeutics.

The pathogenesis of RA is significantly influenced by the gut microbiota (Chen Y. et al., 2022). Dysbiosis of the gut microbiota can cause RA and other inflammatory diseases, and short-chain fatty acids (SCFAs) are important components of the gut environment. Several studies (Lin et al., 2023; Guan et al., 2025) have shown that imbalances in the gut microbiota and SCFAs can lead to dysregulation of immune cells and damage to the gut barrier and joints, which can have serious implications for the body’s health. SCFAs can bind to specific receptor proteins and regulate T cell activation, thereby inhibiting T cell-mediated autoimmune responses (Li J. S. et al., 2022), suppressing RA symptoms and maintaining the health of the body.

Metabolomics explores the relationship between endogenous metabolites and diseases through qualitative or quantitative analysis, providing an effective method to discover biomarkers for disease diagnosis and explore the mechanisms of drug therapy (Xu et al., 2022). Specifically, metabolomics analyses evaluate all metabolites in the sample, which is consistent with the holistic approach of traditional Chinese medicine therapy. Therefore, the integration of metabolomics and 16S rRNA sequencing technology can analyze the relationship between drugs, metabolites and RA from a holistic perspective (Wang et al., 2025), which can help to explore the mechanism of action of drugs in the treatment of RA and find better therapeutic approaches.

Complete Freund’s Adjuvant (CFA) is an immune booster consisting of mineral oil, emulsifier and inactivated *Mycobacterium* that significantly activates cell-mediated and immune responses *in vivo* (Holmdahl et al., 2001). Studies have shown that the CFA-induced adjuvant arthritis model closely resembles human RA in terms of pathological features such as synovial hyperplasia, inflammatory cell infiltration and bone erosion (Snekhalatha et al., 2013). It is one of the best experimental models to study the role of RA in disease.

*Xanthii fructus* (Chinese name Cang Er Zi) is the dry mature fruit with involucre of *Xanthium sibiricum* Patr. in the family of Asteraceae, which was first recorded in *Shennong*’*s Herbal Classic* of the Materia Medica, and is widely distributed in the northeast of China (Chinese Pharmacopoeia Commission, 2020). According to *Shennong*’*s Herbal Classic*, its stir-baked product (stir-baked *Xanthii fructus*, XF) is applied in treating wind-cold, dredging nasal cavities, and relieve joint swelling and pain (Wu, 1982). It is recorded in *the Essential of Materia Medica* that XF can treat rheumatism and Bì syndrome after being roasted (Li, 1975). Modern pharmacological studies (Huang et al., 2011; Park et al., 2015; Xiong et al., 2025) have shown that XF has anti-inflammatory and analgesic effects, can reduce the level of inflammatory factors, reduce joint edema, etc., and significantly improve the onset of RA (Jiang et al., 2023; Zhang et al., 2023); it has also been confirmed in the domestic clinical studies (Yu, 2005) that XF has good therapeutic effects in the treatment of RA, and it can effectively alleviate joint pain and tissue edema, which makes it an effective drug for the treatment of RA, whereas, its mechanism of action requires further study. The present study focused on the changes in metabolites and gut microbiota after XF intervention in AA rats, and the affected microbiota, metabolites and SCFAs were comprehensively analyzed to provide a certain basis for further research on the application of XF in RA.

## Materials and methods

### Materials and reagents

CFA (batch no. SLCN3573) was purchased from Sigma-Aldrich (Shanghai) Trading Co., Ltd. Methotrexate Tablets (batch no. 97221004) were bought from Shanghai Sine Pharmaceutical Co., Ltd. Formic acid, methanol and acetonitrile were obtained from Thermo Fisher Scientific Inc. and deionized water was supplied by Guangzhou Watson’s Food and Beverage Co., Ltd.

### Preparation and composition identification of XF ethanol extract

XF (batch no. 20230223) was acquired from Beijing TongRen Tang, complies with the standards of the Chinese Pharmacopeia (2020 edition), and identified by Associate Professor Li Ke (School of Pharmacy, Shandong First Medical University) as XF. XF was extracted by the water bath reflux method. XF was ground into powder and added to 8 times 75% ethanol, then extracted for 2 h. Collect the filtrate and repeat the aforementioned steps with the filter residue. The filtrate was again collected, and the residue was added to 6 times 75% ethanol and extracted again for 1 h. All filtrates were combined and concentrated to obtain XF concentrate, which was stored in –80°C refrigerator for spare use.

Precisely, 2 g of XF concentrate was diluted to 0.1 g/mL with 50% methanol. The XF solution was centrifuged (12,000 rpm, 10 min), the supernatant removed and filtered. A Vanquish Flex UPLC system coupled to a Q Exactive MS system was utilized for the UPLC-MS analyses. Scanning in positive and negative ion modes (UPLC-MS conditions are described in Supplementary File).

### Animal

Healthy male SPF grade SD rats (180-200 g) were acquired from Shandong Jinan Pengyue Laboratory Animal Breeding Co., Ltd. (production license No. SCXK (Lu) 20220006). All rats were housed in SPF-grade chambers with a temperature of 24 ± 2°C, a relative humidity of 60 ± 5%, and a 12-h light-dark cycle. All animal procedures were approved by the Animal Ethics Committee of Shandong First Medical University (approval No. SYXK (Lu) 20190022).

### AA model induction and drug treatment

All rats were randomly assigned to an average of 5 groups after 1-week adaptive feeding, including the sham-operated (SHA) group, the AA model (AAM) group, the methotrexate (MTX) group, the low-dose XF (XFL) group, and the high-dose XF (XFH) group (*n* = 10). Rats in the AAM, MTX, XFL and XFH groups were immunized by subcutaneous injection of 0.1 mL of complete Freund’s adjuvant into the right hind paw, and 0.1 mL saline was injected into the SHA group. Drug equivalency conversions were performed based on human and rat body surface area to calculate the amount of drug to be administered to rats. The MTX group were treated with 0.9 mg/kg MTX twice a week. The XFL group were garaged with 0.9 g/kg XF and the XFH group were garaged with 1.8 g/kg XF, while the SHA and AAM groups received the same dose of pure water daily once for 5 weeks.

### Physiological indicators in rats

Before the modeling, body weight and right hindpaw volume were measured in all rats. After establishing the rat model, these parameters and arthritis index (AI) were measured weekly in all rats. The AI scoring criteria are as follows: 0, no symptoms; 1, toe joint erythema or swelling; 2, toe and toe joint swelling; 3, toe and ankle joint swelling; and 4, whole paw swelling and ankle joint swelling. A total AI ≥ 4 was considered successful for modeling, with a total AI ≤ 8 per rat. The spleens were excised and weighed for analysis, and spleen index was calculated for each rat.

Spleen index = wet weight of spleen (mg)/body weight of rats (g)

### Sample collection and pre-treatment

Following 5-week administration, rats were euthanized for sample collection. Blood samples were left to stand and centrifuged (4°C, 3,000 rpm, 10 min), the supernatant was aspirated and stored at –80°C. Colon and ankle joint tissues were fixed in 4% paraformaldehyde.

### Serum biochemical analysis

Serum TNF-α, IL-6, IL-8, IL-17, IL-4, IL-10 levels were quantified by ELISA kits and OD values measured with microplate marker.

### Histological assessment

The colon and ankle joint sections were stained by hematoxylin-eosin stain method. Immunohistochemical staining was performed to assess expression levels of matrix metalloproteinase (MMP)-2, vascular endothelial growth factor (VEGF), nuclear factor kappa-B (NF-κB), and zonula occludens-1 (ZO-1) (Immunohistochemical staining methods are described in Supplementary File).

### Serum metabolomics analysis

Serum samples were separated by UPLC and analyzed by mass spectrometry with Xevo G2-XS Q/TOF mass spectrometer in positive and negative ion modes (UPLC-MS conditions are described in Supplementary File).

### 16S rRNA high-throughput sequencing

Fecal DNA was extracted using cetyltrimethylammonium bromide method, with V4 region (515F and 806R) amplification. Libraries were prepared using NEB Next^®^ Ultra DNA Library Prep Kit and sequenced on NovaSeq 6000. After sequencing, the QIIME software package was used to perform quality filtering, trimming, de-noising, merging, and chimera removal. Then, α-diversity analysis and β-diversity analysis were performed using the Qiime2 diversity plugin.

### SCFAs content determination

Agilent 7890B GC system coupled with 5977B MSD system were used to determine the content of SCFAs in colon contents. Mass spectral scans were performed in SCAN/SIM mode (GC-MS conditions are described in Supplementary File).

### Data analysis

GraphPad Prism 8 software was used for visualization and IBM SPSS Statistics 26 for significance analysis. Metabolomics data were analyzed with Progenesis QI and EZinfo 2.0. Differential metabolites were screened using VIP > 1.0 and *p* < 0.05 obtained from the OPLS-DA model, and the model quality was evaluated by R^2^Y and Q^2^. The HMDB database was used to annotate the differential metabolites and pathways.

## Results

### Identification of components in XF

**FIGURE 1:**
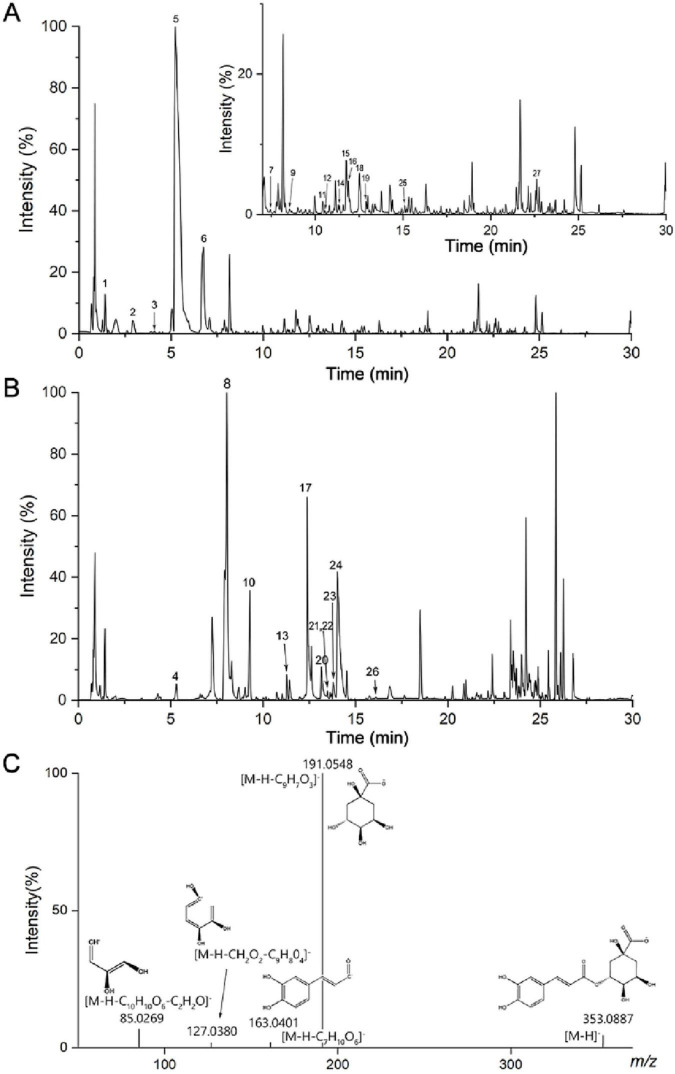
Base peak ion diagram of XF extract in **(A)** positive and **(B)** negative ion mode, **(C)** mass spectrometry of chlorogenic acid in negative ion mode.

Figures 1a,b shows the base peak ion diagram in positive and negative ion modes. The substances have a good separation degree and the system was stable under these conditions. In the negative ion mode, the ion peak m/z was 353.0880 and the retention time was 8.01, as shown in Figure 1C. Its fragment ions were 191.0548, 163.0401, 127.0380, 85.0269, [M-H-C_9_H_7_O_3_]^–^, [M-H-C_7_H_10_O_6_]^–^, [M-H-CH_2_O_2_-C_9_H_8_O_4_]^–^, [M-H-C_10_H_10_O_6_-C_2_H_2_O]^–^, respectively. Further confirmation was achieved by comparing with standards and database entries, identifying the compound as chlorogenic acid. Finally, a total of 27 components were identified (Supplementary Table 1), including protocatechuic acid, neochlorogenic acid, protocatechuic aldehyde, caffeic acid, and chlorogenic acid, etc.

### Therapeutic effects of XF on AA rats

**FIGURE 2:**
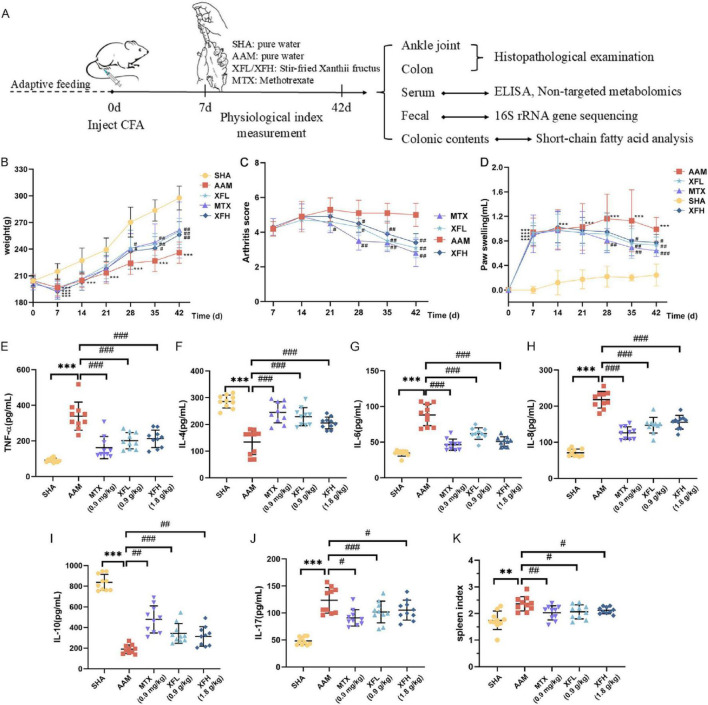
**(A)** experimental design scheme, **(B)** weight change of rats in each group, **(C)** arthritis score, **(D)** foot volume change, **(E–J)** serum inflammatory factor levels and **(K)** spleen index. Dosage: MTX, 0.9 mg/kg; XFL, 0.9 g/kg; XFH, 1.8 g/kg. Data are presented as mean ± SD. Significance: *p < 0.05, **p < 0.01, ***p < 0.001 vs. SHA group; ^#^*p* < 0.05, ^##^*p* < 0.01, ^###^*p* < 0.001 vs. AAM group.

During the experiment, body weight, foot volume and AI changes were recorded for each group of rats. The results (Figures 2A–D) revealed no significant differences (*p* > 0.05) in body weight, foot volume, and AI of rats in the SHA, AAM, XFL, XFH, and MTX groups before modeling. On the 7th day, rats from the AAM, XFL, XFH, and MTX groups showed significantly (*p* < 0.01) larger weight loss, increased foot swelling, and increased compared to the SHA group. After treatment, the rats in the XFL, XFH, and MTX groups showed accelerated weight gain, reduced foot swelling and decreased AI, which was different from the AAM group, moreover, the rats’ body weights, foot swelling, and AI returned to normal. Furthermore, the biochemical results (Figures 2E–K) showed that the spleen index and serum levels of TNF-α, IL-6, IL-8, and IL-17 were elevated (*p* < 0.01), and these levels were reduced in the XFL, XFH and MTX groups after treatment (*p* < 0.05). The levels of the anti-inflammatory factors IL-4 and IL-10 were significantly reduced in the AAM group of rats (*p* < 0.01), and increased in the XFL, XFH, and MTX groups after treatment (*p* < 0.01).

### XF improves intestinal barrier function and ankle joint destruction in AA rats

**FIGURE 3:**
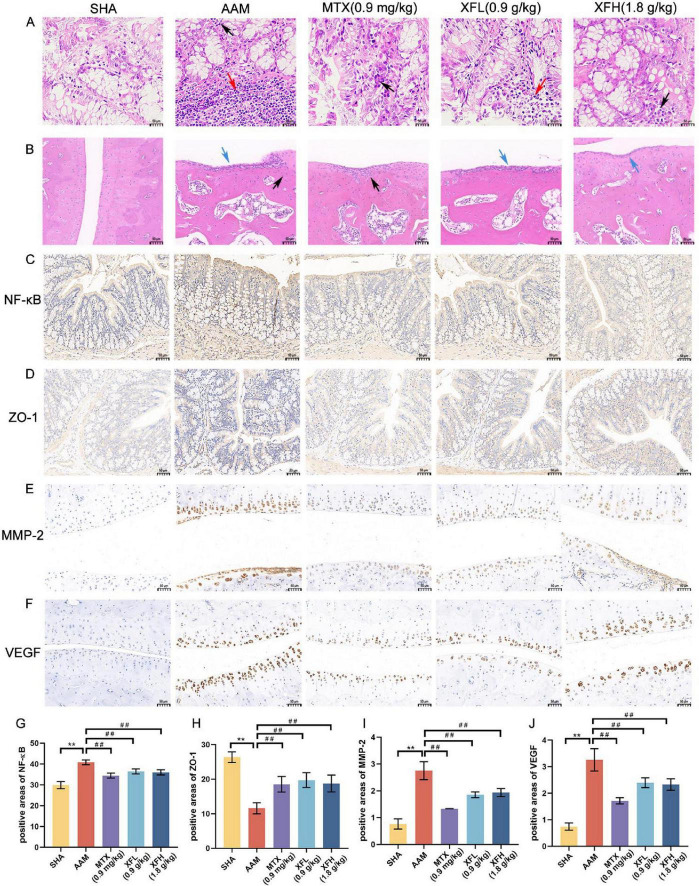
Different groups of rats **(A)** colon tissue and **(B)** ankle joint hematoxylin-eosin staining image, immunohistochemical expression image and its positive area comparison. The expression images of **(C)** NF-κB and **(D)** ZO-1 in colon tissue, the expression images of **(E)** MMP-2 and **(F)** VEGF in ankle joint tissue, and the positive expression areas of **(G–J)** NF-κB, ZO-1, MMP-2 and VEGF were compared. Dosage: MTX, 0.9 mg/kg; XFL, 0.9 g/kg; XFH, 1.8 g/kg. Scale = 50μm, data expressed as mean ± SD (*n* = 4). Significance: **p < 0.01 vs. SHA group; ^##^*p* < 0.01 vs. AAM group.

As Figures 3a,b shows, the rats in the AAM group developed gut inflammation with inflammatory cell infiltration in the colon tissue (black arrows) and visible lymph nodes in the submucosa (red arrows). The ankle joints exhibited structural damage and inflammatory cell infiltration (black arrows) with connective tissue hyperplasia (blue arrows), which were consistent with severe AA symptoms. Following treatment, the inflammatory symptoms of XFL, XFH, and MTX group were improved to different degrees, with less inflammatory cell infiltration and no obvious connective tissue hyperplasia.

Immunohistochemical results revealed (Figures 3C–J) a significantly increased area of NF-κB positivity in AA rats’ colonic tissues (*p* < 0.01), with darker tan staining, and the level of ZO-1 was reduced (*p* < 0.01), displaying a lighter than staining, indicating intestinal damage. Following treatment, the expression of NF-κB was reduced (*p* < 0.01), while the level of ZO-1 was increased (*p* < 0.01) in the rats of the XFL, XFH, and MTX groups. These findings indicate that XFL, XFH and MTX can inhibit intestinal inflammation and protect the gut barrier integrity in rats by regulating the NF-κB signaling pathway and enhancing the expression of tight junction proteins. Synovial MMP-2 and VEGF expression was significantly increased in AA rats (*p* < 0.01), showing a deeper brown color staining. Following treatment, the content of MMP-2 and VEGF in the XFL, XFH, and MTX groups was reduced (*p* < 0.01).

### XF induces changes in AA rat serum endogenous substances

**FIGURE 4:**
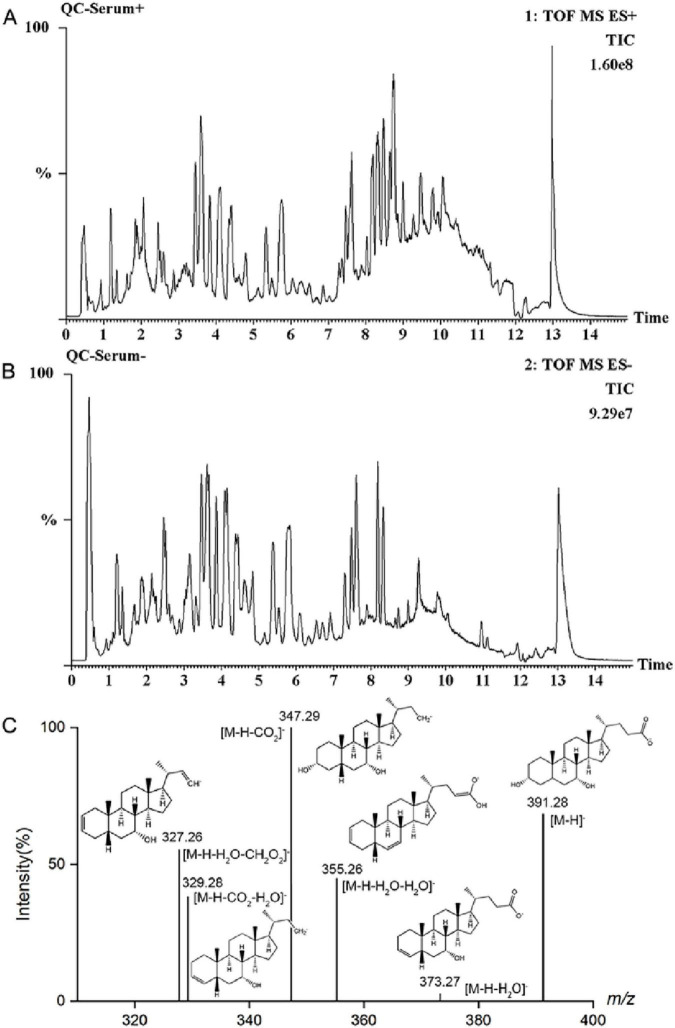
TIC diagram of serum QC sample in **(A)** positive ion and **(B)** negative ion mode, **(C)** mass spectrometry of CDCA in negative ion mode.

**FIGURE 5:**
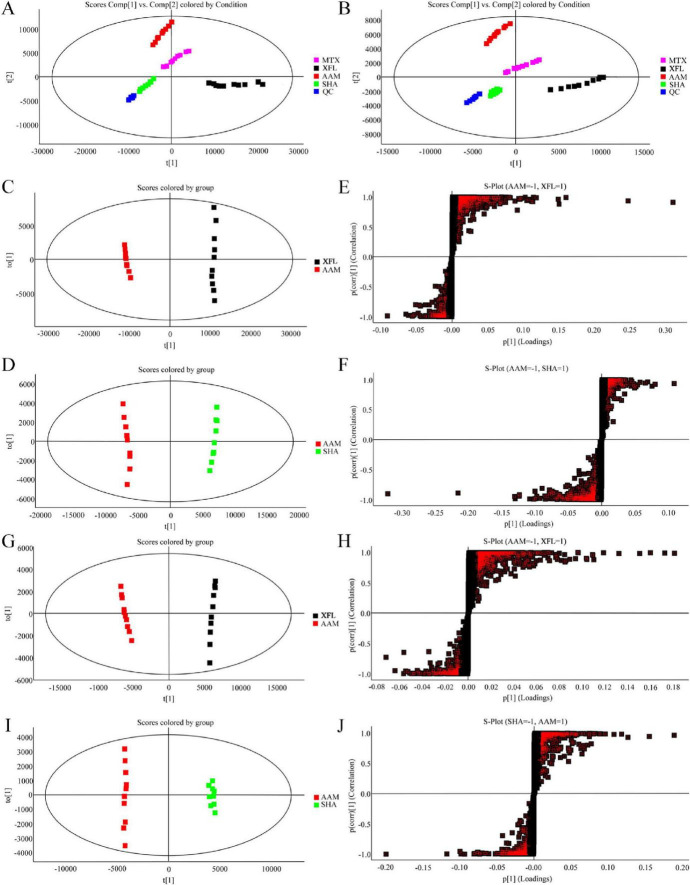
Effect of XF on serum metabolic profile of AA rats. PCA score plots in **(A)** positive ion mode and **(B)** negative ion mode. OPLS-DA score plot and S-Plot in positive ion mode **(C–F)** and negative ion mode **(G–J)**. Dosage: MTX, 0.9 mg/kg; XFL, 0.9 g/kg.

By physiological indexes and histopathological analysis, the XFL group showed a better therapeutic effect than the XFH group. Therefore, 0.9 g/kg XF was chosen as the dose for the follow-up study. Total ion chromatogram (TIC) of QC serum samples in positive and negative ion modes (Figures 4a,b) showed good separation and systemic stability of each metabolite. The R^2^Y and Q^2^ values of the PCA, OPLS-DA, and S-plot analysis results (Figure 5) are 99%, indicating that the model is stable and reliable, with high within-group aggregation and between-group separation. The compounds marked in red indicated potential biomarkers, which were imported into Progenesis QI for further screening and identification.

**FIGURE 6:**
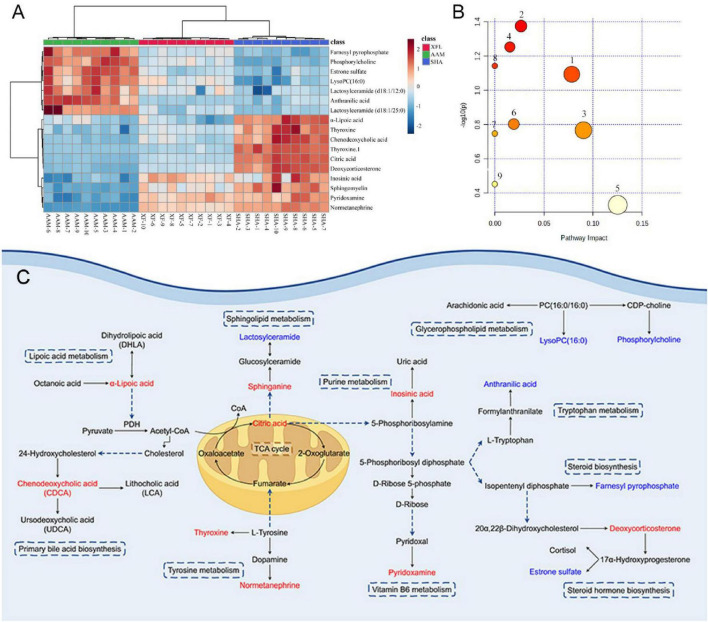
**(A)** Heat map visualizing the relative abundance of serum metabolites. **(B)** Analysis of metabolic pathways of serum metabolites affected by AA. **(C)** Metabolic network map of endogenous metabolites after XF treatment. Red represents elevated level; blue represents reduced levels. 1. Vitamin B_6_ metabolism, 2. Glycerophospholipid metabolism, 3. TCA cycle, 4. Tyrosine metabolism, 5. Purine metabolism, 6. Steroid hormone biosynthesis, 7. Sphingolipid metabolism, 8. Lipoic acid metabolism, 9. Primary bile acid biosynthesis.

As an example (Figure 4C), the metabolite at m/z 391.28 in negative ion mode was detected at the [M-H]^–^ precursor ion at 1.76 min. Fragment ions [M-H-H_2_O]^–^, [M-H-H_2_O-H_2_O]^–^, [M-H-CO_2_]^–^, [M-H-CO_2_-H_2_O]^–^, and [M-H-H_2_O-CH_2_O_2_]^–^ were respectively detected at m/z 373.27, 355.26, 347.29, 329.28, and 327.26. Further confirmation was achieved by comparing with standards and database entries, and the compound was identified as chenodeoxycholic acid (CDCA). Finally, a total of 17 endogenous metabolites (Supplementary Table 2) were analyzed, and 9 metabolic pathways were enriched by MetaboAnalyst visualization (Figures 6a,b). There were changes in 17 endogenous metabolites in the AAM group in comparison with SHA, of which 10 were downregulated and 7 were upregulated. After XF treatment, some of the AA-induced endogenous metabolite alterations were reversed, with 4 upregulated endogenous metabolites and 7 downregulated endogenous metabolites. In addition, we determine the effects of XFL on the biometabolic pathways and metabolic processes of serum endogenous metabolites in AA rats (Figure 6C).

### XF affects the composition and make-up of gut microbiota

**FIGURE 7:**
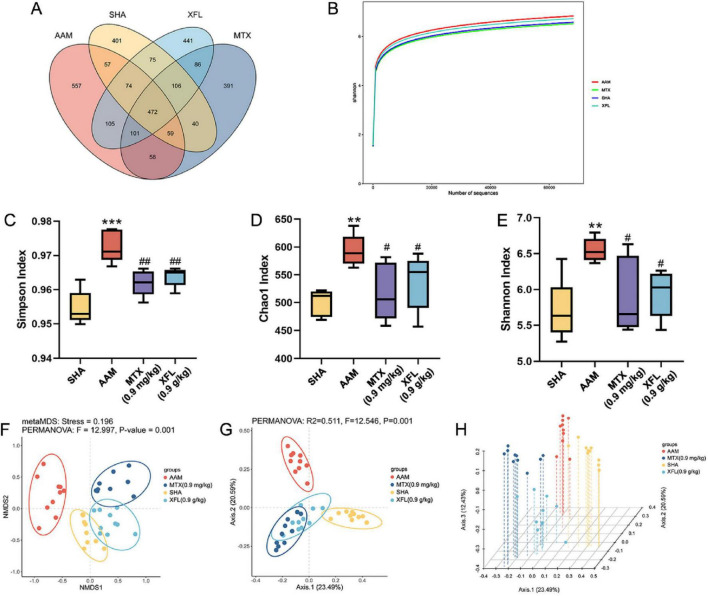
Effects of XF and methotrexate on intestinal flora in AA rats. **(A)** Venn, **(B)** rarefaction curve: Shannon, **(C)** Simpson index, **(D)** Chao1 index, **(E)** Shannon index, **(F)** NMDS, **(G)** PCoA, **(H)** PCoA 3D. Dosage: MTX, 0.9 mg/kg; XFL, 0.9 g/kg. Data expressed as mean ± SD. Significance: **p < 0.01, ***p < 0.001 vs. SHA group; ^#^*p* < 0.05, ^##^*p* < 0.01 vs. AAM group.

To determine the effect of XF on the gut flora of AA rats, we used 16S rRNA high-throughput sequencing to study the changes in gut flora between groups. The Venn diagram (Figure 7A) showed that the structure of the gut flora was altered in AA rats and that the XF intervention had an effect on the rats in the AAM group. The rarefaction curves (Figure 7B) show that there is sufficient sample volume and a reasonable amount of sequencing data for the subsequent analyses. The results (Figures 7C–E) found that the Shannon index, Chao1 index, Simpson index and Simpson index were increased in AA rats (*p* < 0.01), but these indexes in XFL and MTX groups were lower (*p* < 0.05) and tended to the SHA group after drug administration. Meanwhile, principal coordinate analysis (PCoA) and non-metric multidimensional scaling (NMDS) revealed differences in each group (Figures 7F–H).

**FIGURE 8:**
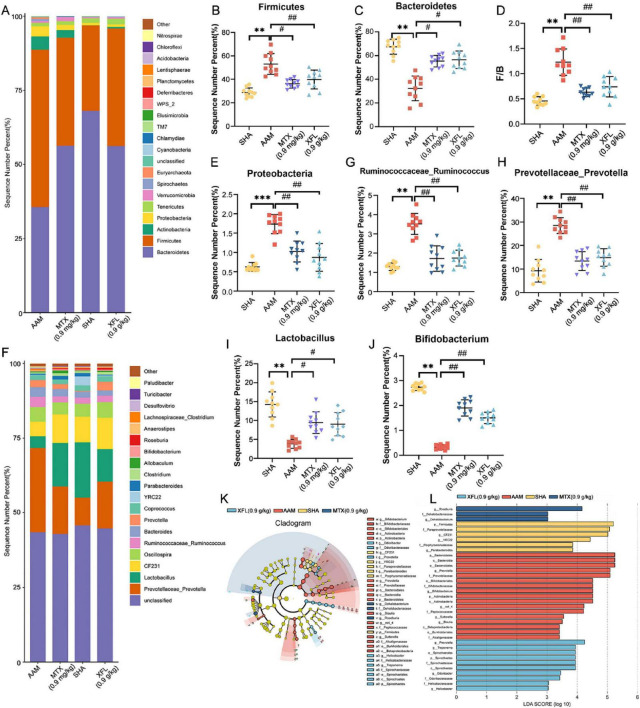
XF treatment improves gut microbiota imbalance in AA rats. **(A)** Phylum horizontal stacking histogram, **(B)** Firmicutes and **(C)** Bacteroidetes relative abundance, **(D)** F/B ratio, **(E)** Proteobacteria relative abundance, **(F)** genus horizontal stacking histogram, **(G)** *Ruminococcus*, **(H)** *Prevotella*, **(I)** *Lactobacillus* and **(J)** *Bifidobacterium* relative abundance, **(K)** LEfSe analysis and **(L)** LDA > 2 taxa. Dosage: MTX, 0.9 mg/kg; XFL, 0.9 g/kg. Data expressed as mean ± SD. Significance: *p < 0.05, **p < 0.01 vs. SHA group; ^#^*p* < 0.05, ^##^*p* < 0.01 vs. AAM group.

At the phylum level (Figures 8A–E), Firmicutes and Bacteroidetes were the dominant phyla. Compared to the SHA group, the AAM group showed a decreased abundance of Bacteroidetes (*p* < 0.01), while Firmicutes and Proteobacteria were increased (*p* < 0.01), resulting in significantly higher F/B values (*p* < 0.01). After treatment, the abundance of Bacteroidetes increased (*p* < 0.05) and the abundance of Firmicutes and Proteobacteria decreased (*p* < 0.05), leading to a reduction in F/B values (*p* < 0.05) in the XFL and MTX groups. At the genus level (Figures 8F–J), the abundance of *Prevotella* and *Rumatococcus* was increased in the AAM group (*p* < 0.01), whereas the abundance of *Lactobacillus* and *Bifidobacterium* was decreased (*p* < 0.01). Following treatment, the XFL and MTX groups showed different degrees of reversal. Furthermore, the composition of gut microbiota was identified at the genus level by using LDA and LEfSe. A total of 36 microbiota with differences between groups were screened (Figures 8K,L). Among them, the SHA, AAM, XFL, and MTX groups had 6, 17, 10 and 3 significantly enriched microbiotas, respectively.

### XF affects the level of SCFAs

**FIGURE 9:**
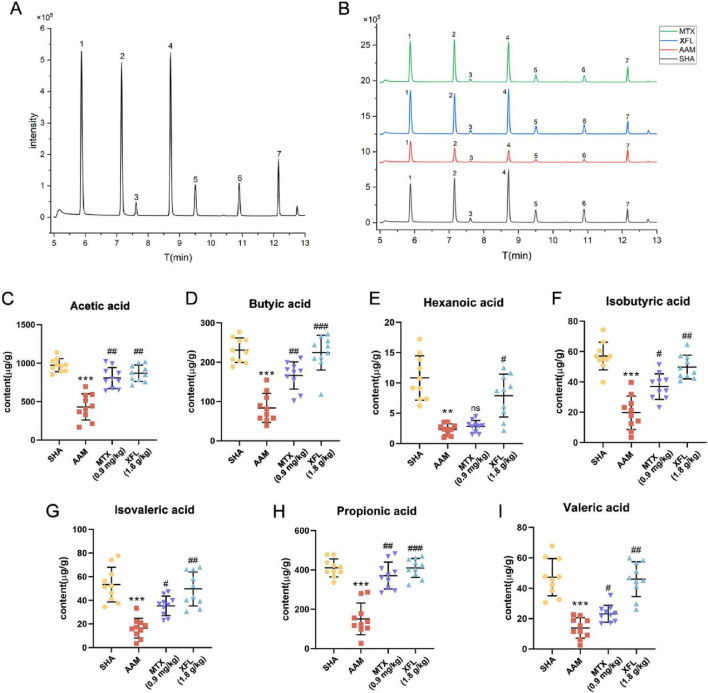
Effect of XF on colon contents SCFAs in AA rats. Total ion chromatogram of the **(A)** QC and **(B)** rat sample, contents of **(C)** acetic acid, **(D)** butyric acid, **(E)** hexanoic acid, **(F)** isobutyric acid, **(G)** isovaleric acid, **(H)** propionic acid, **(I)** valeric acid. Dosage: MTX, 0.9 mg/kg; XFL, 0.9 g/kg. Data expressed as mean ± SD. Significance: **p < 0.01, ***p < 0.001 vs. SHA group; ^#^*p* < 0.05, ^##^*p* < 0.01, ^###^*p* < 0.001 vs. AAM group. 1. acetic acid, 2. propionic acid, 3. isobutyric acid, 4. butyric acid, 5. isovaleric acid, 6. valeric acid, 7. hexanoic acid.

Figures 9a,b show the total ion chromatogram obtained from QC samples and rats’ samples from different groups analyzed by GC-MS, respectively. The total ion chromatogram was similar between the different samples, which indicated that the method was stable and reproducible and could be used for subsequent analyses. As shown in Figures 9C–I, the content of SCFAs in the colonic contents of rats in the AAM group was reduced compared with those in SHA group (*p* < 0.01). Following treatment, the levels in rats in the XFL and MTX groups were increased (*p* < 0.05) and tended to be closer to those of healthy rats.

### Strong correlation between gut microbiota and serum metabolites, SCFAs

**FIGURE 10:**
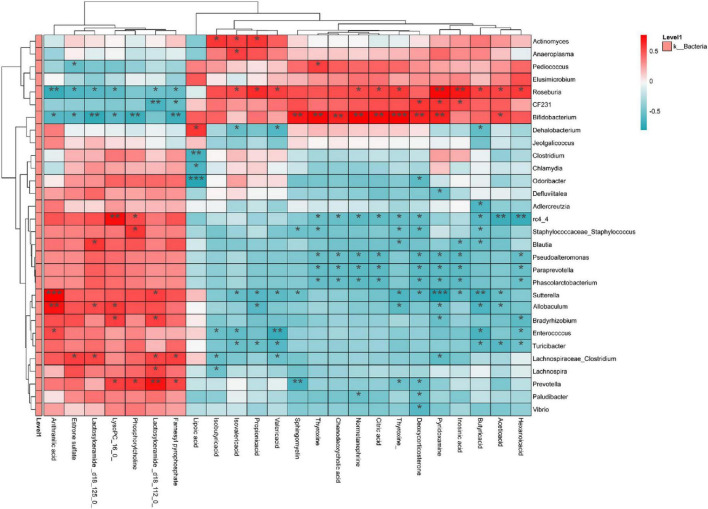
Analysis of correlations between gut microbiota on genus level and serum metabolites and SCFAs. Red indicates positive correlation; blue indicates negative correlation. Significance: *p < 0.05, **p < 0.01, ***p < 0.001.

Spearman correlation analyses of microbiotas with significant differences at the genus level with SCFAs and serum metabolites were performed to explain the relationship between the three (Figure 10). *Roseburia* was positively correlated with inosinic acid, thyroxine, sphingomyelin, pyridoxamine, citric acid, normetanephrine (NE) and deoxycorticosterone, and negatively correlated with lactosylceramide, estrone sulfate, anthranilic acid, lysoPC, phosphatidylcholine (PC), and farnesyl pyrophosphate (FPP). *Bifidobacterium* was positively correlated with lactosylceramide, estrone sulfate, PC, lysoPC, and FPP, and negatively correlated with inosinic acid, thyroxine, sphingomyelin, pyridoxamine, citric acid, NE, deoxycorticosterone and α-Lipoic acid (α-LA). *Prevotella* was positively correlated with lactosylceramide, estrone sulfate, anthranilic acid and PC, and negatively correlated with thyroxine. Furthermore, SCFAs showed a positive correlation with *Roseburia* and *Actinomyces*, while a negative correlation with *Sutterella*, *Enterococcus*, *Allobaculum*, and *Turicibacter*.

## Discussion

RA is manifested by synovitis caused by metabolic pathways disorder (Xu et al., 2022). It affects the body’s energy conversion, inflammatory factor levels, joint destruction and immune response. Recent studies have found that abnormal immune responses are closely linked to the pathogenesis of RA, with increased secretion of pro-inflammatory cytokines (e.g., TNF-α, IL-1β, and IL-6 etc.) leads to an imbalance of Treg/Th17 cells (Liu et al., 2023; Bhutta et al., 2024), which exacerbates the development of inflammation; concurrently, these pro-inflammatory cytokines activate a number of pathways involved in bone destruction (Chen Q. Y. et al., 2022), affecting the balance of osteoclasts and osteoblasts, resulting in bone destruction. XF can be traced back to *Shennong*’*s Herbal Classic*, which can help treat wind-cold, dredge the nasal cavity and dispel wind-cold (Wu, 1982). Research shows that the sesquiterpene lactones in XF effectively inhibit the phosphorylation process of AKT and mTOR, and inhibit the expression of the PI3K/AKY/mTOR signaling pathway, leading to the inhibition of pro-inflammatory cytokine expression (Wang et al., 2023). Phenolic acid compounds have been shown to down-regulate the expression of Toll-like receptors and inhibit the activation of the NF-κB signaling pathway, thereby reducing oxidative stress *in vivo* (Jiang et al., 2018). Our previous research demonstrated that XF possesses anti-inflammatory properties.

Studies have shown that overexpression of MMP-2 and VEGF *in vivo* induces an imbalance in matrix protein synthesis and degradation, and aberrant expression by macrophages and fibroblasts, which in turn induces angiogenesis and bone destruction, exacerbating RA symptoms in patients (Taylor, 2002; Meng et al., 2023). When NF-κB is stimulated or activated, it promotes the expression of various pro-inflammatory factors, such as TNF-α, and induces intestinal barrier disruption and inflammatory cascade responses, whereas ZO-1 regulates the balance of inflammatory factors and microbiota in the gut and exerts a protective effect on the intestinal barrier (Li D. et al., 2022; Yuan et al., 2023). The physiological and pathological experiments in this study showed the same trend as the above studies, and the results showed low levels of pro-inflammatory factors in rat serum, with reduced joint swelling and intestinal inflammation after drug treatment. This suggests that XF can protect articular cartilage and subchondral bone by regulating the level of immunity *in vivo*, restoring the balance between synthesis and degradation of MMPs in the joints, and inhibiting abnormal neoangiogenesis. Meanwhile, 16S rRNA high-throughput sequencing, CG-MS analysis and serum metabolomics studies revealed that XF could also inhibit the inflammatory response in the gut, regulate the level of SCFAs and repair the intestinal barrier function, thereby exerting therapeutic effects through the gut-joint axis, providing strong evidence for the *in vivo* mechanism of action of XF in alleviating AA.

The gut microbiota affects metabolic regulation, gut mucosal barrier function and immune function, and its disruption is closely linked to the onset and development of RA. The results revealed significant alterations in in AA rat gut structure. XF could reverse this change in AA rats, and bring them closer to the SHA group. Bacteroidetes and Firmicutes, the dominant gut flora, play an essential role in the regulation of the immune response. Bacteroidetes can induce over-expression of anti-citrullinated peptide antibody by promoting the conversion of arginine to citrulline to increase vascular permeability of the synovial membrane and to activate mesenchymal cells in the joint to destroy articular cartilage (De Luca and Shoenfeld, 2019). Proteobacteria, a major gram-negative bacillus, induces immune disorders and enhance the release of inflammatory factors (Yang and Cong, 2021). Firmicutes have a crucial role in metabolic processes by promoting the synthesis of SCFAs. SCFAs promote the production of anti-inflammatory cytokines, which exert immunomodulatory effects and inhibit excessive inflammatory responses, thereby helping to maintain of intestinal immune homeostasis and systemic immune balance (Yu, 2005). *Prevotella* can affect Th17 and Treg cell-mediated immune responses, resulting in IL-17 accumulation in the joints and exacerbating joint swelling and pain (De Luca and Shoenfeld, 2019). *Lactobacillus* and *Bifidobacterium*, as beneficial bacteria in the gut, can inhibit the proliferation of harmful bacteria and participate in immunomodulation, promoting the production of antibodies and interferon and improving immunity (Luo et al., 2025). This study revealed a trend in gut microbiota similar to that observed in previous reports, suggesting that XF could achieve anti-inflammatory effects by altering the composition of the gut microbiota in AA rats, influencing the immune response and reducing inflammatory factors release.

Furthermore, the gut microbiota has a greater influence on the regulation of the metabolic in AA rats. Therefore, the endogenous metabolites in serum samples from AA rats after XF treatment were analyzed by UPLC-Q-TOF/MS. The results showed that XF treatment effectively reversed some of the AA-induced endogenous metabolite alterations, with up-regulation of pyridoxamine, inosinic acid, sphingomyelin, thyroxine, NE, α-LA, citric acid, CDCA and deoxycorticosterone levels, and down-regulation of estrone sulfate, lysoPC (16:0), lactosylceramide (d18:1/12:0), anthranilic acid, FPP, PC and Lactosylceramide (d18:1/25:0) levels. Moreover, the metabolic pathways involved included vitamin B6 metabolism, tryptophan metabolism, glycerophospholipid metabolism, steroid hormone biosynthesis, lipoic acid metabolism, tyrosine metabolism, TCA cycle, and primary bile acid biosynthesis.

Pyridoxamine is an analog of vitamin B6 that exists in the body primarily as a phosphate ester. Pyridoxamine has been shown to inhibit M1 macrophage polarization, suppress NF-κB expression, and reduce the levels of inflammatory factors in visceral and perivascular adipose tissue (Oh et al., 2019). In this experiment, pyridoxamine levels in AA rats were significantly down-regulated and vitamin B6 metabolism was inhibited, whereas pyridoxamine levels were significantly increased after XF treatment, suggesting that XF can interfere with vitamin B6 metabolism.

Lactosylceramide and PC are both involved in glycerophospholipid metabolism. Lactose ceramide, as a bioactive lipid, plays a role in inducing the expression of inflammatory factors and angiogenesis (Mu et al., 2009). PC is involved in the production of LysoPC and plays an important role in regulating cellular lipid metabolism and homeostasis. In this experiment, significantly increased levels of lactosylceramide and PC were found in the serum of rats in the AAM group, whereas the levels of lactosylceramide and PC in the serum of AA rats treated with XF were significantly decreased. These findings suggest that XF can regulate glycerophospholipid metabolism in AA rats and ameliorate the inflammation of AA.

As one of the products of tryptophan metabolism, anthranilic acid is produced from kynurenine under the catalysis of kynureninase (Pawlowski et al., 2021), participating in protein synthesis and the body’s immune response. Increased anthranilic acid levels are observed in RA (Igari et al., 1987). This study suggests that RA leads to disorders of tryptophan metabolism and that XF treatment modulates the tryptophan metabolic pathway and significantly reduces anthranilic acid levels.

FPP is an intermediate in the mevalonate pathway that can be transformed into geranyl pyrophosphate or squalene, which activates immune cells and leads to inflammation (Wang and Ye, 2015). In conditions such as injury, inflammation, cancer and immune cell activation, the metabolic state of cells changes, promoting the accumulation of terpenes. In environments conducive to FPP production and release, the substance transmits signals to surrounding tissues and cells, thereby triggering morbidity associated with cell death (Gruenbacher and Thurnher, 2017). In the present study, XF significantly downregulated FPP levels in the serum of AA rats, reduced inflammation and modulated steroid hormone biosynthesis.

Thyroxine and NE are both produced by tyrosine metabolism and are closely related to the development of RA (Lorton and Bellinger, 2015). Under inflammatory conditions, pro-inflammatory factors affect the hypothalamus, causing abnormal thyroid function, which leads to decreased thyroxine and NE secretion, thus affecting the local and systemic immune response (Soy et al., 2007; Bellinger and Lorton, 2014). Treatment with XF resulted in significant recovery of the levels of thyroxine and NE, which promoted metabolism, improved inflammatory responses, and modulated tyrosine metabolic pathways in AA rats.

α-LA is involved in lipoic acid metabolism and is produced by mitochondrial α-lipoyl synthase, exerting anti-inflammatory and anti-oxidant effects (Wang et al., 2016). Reports shown that α-LA inhibits NF-κB activation by TNF-α in RA fibroblast-like cells (Lee et al., 2008). Similarly, AA rats in this study exhibited reduced α-LA levels and increased TNF-α secretion in serum. However, following XF treatment, α-LA levels increased, TNF-α secretion decreased, and the NF-κB pathway was inhibited.

Citric acid is an important metabolite of the TCA cycle which plays a key role in energy production and metabolism of cell (Xie et al., 2019). The inflammatory response leads to a decrease in the body’s energy metabolism capacity by altering intracellular metabolic pathways and regulating energy homeostasis, further exacerbating the metabolic burden. This experiment found that the citric acid levels of rats in the XF group significantly recovered to normal levels, suggesting that XF may regulate the TCA cycle by increasing citric acid levels.

CDCA is the main active ingredient in animal bile. It can inhibit major inflammatory cytokines, enhance anti-inflammatory genes and improve insulin sensitivity. In addition, CDCA can significantly reduce inflammation in osteoarthritis, showing promising therapeutic potential (Yan et al., 2015). This study discovered a significant increase in CDCA after treatment with XF, which inhibited the production of inflammatory cells and enhanced the anti-inflammatory effect. This agrees with the pharmacodynamic study results.

Furthermore, a close link was found between the gut microbiota, SCFAs and serum metabolites. The metabolites (SCFAs and so on) produced by the gut microbiota enter the blood circulation and affect the overall metabolic state of the body. Therefore, a Spearman correlation analysis was applied to the gut microbiota, SCFAs and serum metabolites, indicating a strong correlation among them. Changes in the gut microbiota effectively influence metabolic processes in AA rats, leading to inflammation and bone damage, whereas XF effectively improved the gut microbiota, SCFAs and metabolic dysfunction in AA rats and alleviated arthritis symptoms.

**FIGURE 11:**
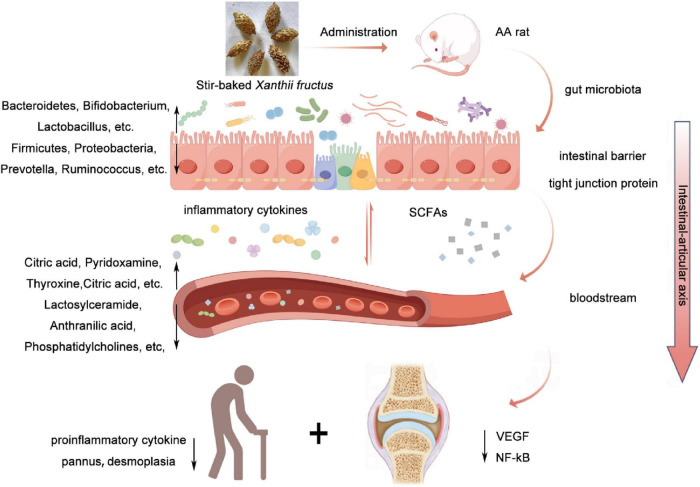
Mechanism of XF in the treatment of AA. XF alleviates AA symptoms by improving immune regulation, oxidative stress, inflammation, intestinal barrier function, intestinal microbiota and metabolic balance.

In this study, XF was found to exert anti-inflammatory, joint protective and immunomodulatory effects in AA rats (Figure 11). In addition, XF exerted anti-inflammatory and joint protective effects by modulating gut microbes and serum metabolites through the gut-joint axis to attenuate inflammatory responses and joint destruction. The results reveal the *in vivo* mechanism of XF in the treatment of RA and its potential in the field of anti-inflammation and joint protection, providing an experimental basis for the application of XF in the treatment of RA.

## Data Availability

The data presented in the study are deposited in the NCBI repository, accession number PRJNA1261398.
