# Supplementary material for: Stir-baked Xanthii fructus ameliorates adjuvant arthritis by regulating gut microbiota, short-chain fatty acids and metabolites
Source: Front Microbiol. 2025 Jun 5;16:1599529. doi: 10.3389/fmicb.2025.1599529 (PMC12178575; doi:10.3389/fmicb.2025.1599529)
Supplement: Supplementary file 1 [file Supplementary_file_1.docx]

Supplementary Material

# Supplementary Data

## LC-MS conditions for component identification analysis.

After sample preparation, the analysis was conducted using a Vanquish Flex UHPLC liquid chromatography system (Thermo Fisher Scientific, USA) connected to a Q Exactive™ mass spectrometer (Thermo Fisher Scientific, USA). The chromatographic separation was performed on an ACQUITY UPLC HSS T3 column (2.1 mm × 100 mm, 1.8 μm). The mobile phases comprised 0.1% formic acid in water (A) and acetonitrile (B). The injection volume was 6 μL, and the flow rate was 0.3 mL/min. The elution gradient of B was as follows: 0-1 min, 2% B; 1-14 min, 2-30% B; 14-25 min, 30-100% B; 25-28 min, 100% B; 28-30 min, 100-2% B. Mass spectrometry scans were performed in positive and negative ion modes: ion source voltages of 3.7 kV and 3.5 kV for positive and negative ions, respectively; capillary heating temperature of 320℃, sheath gas pressure of 30 psi, and auxiliary gas pressure of 10 psi; and solvent heating and evaporation temperature of 300℃, with nitrogen for the sheath, auxiliary, and collision gases.

## Immunohistochemical staining method。

The sections were sequentially rinsed in dewaxing solution and ethanol in different concentration gradients. The sections were then repaired in EDTA (pH 9.0), cooled naturally and placed in PBS (pH 7.4) for washing. The sections were incubated in 3% hydrogen peroxide and then washed in PBS (pH 7.4), and then 3% BSA was added to cover the tissue and closed. Subsequently, primary antibodies (NF-κB, ZO-1, VEGF, MMP-2) were added to the sections individually, incubated overnight and washed in PBS (pH 7.4). The secondary antibody was then added, incubated for 50 minutes and washed with PBS (pH 7.4). Finally, DAB color development solution was added, and after terminating the color development, the sections were retained and counterblued with hematoxylin and dehydrated and sealed. Positive cell expression was observed under an optical microscope and the area of positive expression in the same area was calculated by Image-pro plus 6.0.

## LC-MS conditions and gradient changes for serum metabolomics analysis.

The analysis was performed on a Waters Acquity UPLC liquid chromatography system (Waters, USA) and a Waters Xevo G2-XS Q/TOF mass spectrometer (Waters, USA) using an ACQUITY UPLC BEH C18 column (2.1 mm×50 mm, 1.7 μm); injection volume: 5 μL; flow rate: 0.3 mL/min; the mobile phases comprised 0.1% formic acid in water (A) and acetonitrile (B), and the gradient elution procedure was 0-2 min, 10-58% B; 2-4 min, 58-64% B; 4-6 min, 64-67% B; 6-7 min, 67-85% B; 7-12 min, 85-100% B; 12-13 min, 100-10% B. Data were collected in positive and negative ion modes, respectively; capillary voltage in positive and negative ion modes: 2.5 kV and 2.0 kV, temperature of the ion source: 100℃, temperature of the desolvent gas: 450℃, cone pore voltage of 40 V, nitrogen for the cone pore gas and desolvent gas, and argon for the collision gas.

## CG-MS conditions and gradient changes for short-chain fatty acid analysis.

After sample preparation, analysis was conducted using an Agilent 7890B gas chromatography system connected to a 5977B MSD mass spectrometry system. The separation was performed using a DB-FFAP chromatography column (30 m × 250 µm, 0.25 µm, Agilent), with helium as the carrier gas at a flow rate of 1.0 mL/min with 1 µL per injection, and a split ratio of 10:1. The temperature was increased from 90℃ to 160℃ at 10℃/min, then to 240℃ at 40℃/min and held for 5 min. Mass spectrometry was performed in SCAN/SIM mode: inlet temperature: 250℃, ion source temperature: 230℃, transfer line temperature: 250℃, quadrupole temperature: 150℃, electron bombardment ionisation source: 70 eV.

# Supplementary Tables

## Table S1 Chemical constituents of ethanol extract of XF.

| **No.** | **Retention time (min)** | **Chemical formula** | **Calculated m/z** | **Observe m/z** | **Adduct ion** | **Error (ppm)** | **MS/MS** | **Identificaton** |
| --- | --- | --- | --- | --- | --- | --- | --- | --- |
| 1 | 1.45 | C_5_H_4_N_4_O_2_ | 153.0405 | 153.0407 | M+H | -1.75 | 153.0407, 152.0568, 116.9664, 136.0142, 110.0355 | Xanthine* |
| 2 | 3.18 | C_6_H_6_O_3_ | 109.0290 | 109.0284 | M+H-H_2_O | 4.17 | 95.0500, 87.005, 81.0346, 96.0101, 79.0554 | 5-hydroxymethylfurfural* |
| 3 | 4.53 | C_7_H_6_O_4_ | 155.0336 | 155.0339 | M+H | -2.23 | 137.0234, 113.9642, 131.9744, 72.9384, 90.9486 | Protocatechuic acid* |
| 4 | 5.13 | C_16_H_18_O_9_ | 353.0880 | 353.0878 | M-H | 0.67 | 191.0548, 135.0435, 179.0341, 353.0894, 85.0269 | Neochlorogenic acid* |
| 5 | 5.53 | C_7_H_6_O_3_ | 139.0388 | 139.0390 | M+H | -1.38 | 111.0446, 116.9665, 93.0344, 80.9461, 139.0389 | 3,4-dihydroxybenzaldehyde* |
| 6 | 6.74 | C_9_H_8_O_4_ | 163.0386 | 163.0390 | M+H-H_2_O | -2.24 | 144.0808, 117.034, 89.0395, 99.0448, 143.0339 | 3,4-dihydroxycinnamic acid* |
| 7 | 7.67 | C_8_H_8_O_4_ | 151.0388 | 151.0390 | M+H-H_2_O | -1.01 | 151.0389, 149.0234, 91.0551, 107.0862, 123.0807 | Isovanillic acid* |
| 8 | 8.01 | C_16_H_18_O_9_ | 353.0880 | 353.0878 | M-H | -0.27 | 191.0548, 85.0269, 353.0887, 161.0224, 127.0380 | Chlorogenic acid* |
| 9 | 8.32 | C_25_H_24_O_12_ | 517.1333 | 517.1340 | M+H | 1.14 | 163.0388, 135.0441, 499.1207, 283.0592, 324.0572 | 1,3-dicaffeoylquinic acid* |
| 10 | 9.17 | C_17_H_20_O_9_ | 367.1035 | 367.1034 | M-H | 0.22 | 191.0548, 93.0321, 367.1035, 173.044, 134.0352 | Methyl chlorogenate* |
| 11 | 10.32 | C_10_H_8_O_4_ | 193.0492 | 193.0496 | M+H | -2.02 | 193.0496, 133.0285, 178.0259, 167.9771, 137.0598 | Scopoletin* |
| 12 | 10.37 | C_10_H_10_O_4_ | 195.0647 | 195.0652 | M+H | -2.27 | 195.0651, 177.0545, 194.0637, 131.9743, 113.9641 | Ferulic acid* |
| 13 | 10.95 | C_21_H_20_O_12_ | 463.0888 | 463.0882 | M-H | 1.25 | 300.0277, 271.0249, 255.0295, 243.0296, 151.0015 | Hyperoside* |
| 14 | 11.39 | C_15_H_18_O_3_ | 247.1320 | 247.1329 | M+H | -3.58 | 247.1325, 187.1116, 229.1223, 201.1272, 165.091 | Xanthatin* |
| 15 | 11.76 | C_16_H_18_O_9_ | 355.1012 | 355.1024 | M+H | -3.46 | 163.0389, 164.0423, 135.0441, 117.034 | Cryptochlorogenic acid* |
| 16 | 11.96 | C_10_H_10_O_3_ | 179.0697 | 179.0703 | M+H | -3.25 | 147.044, 179.0704, 161.0596, 119.0497, 133.0648 | Ferulaldehyde* |
| 17 | 12.55 | C_25_H_24_O_12_ | 515.1193 | 515.1195 | M-H | -0.35 | 173.0439, 179.0335, 135.043, 191.0547, 353.0881,127.0379 | 3,5-dicaffeoylquinic acid* |
| 18 | 12.67 | C_16_H_18_O_9_ | 355.1012 | 355.1024 | M+H | -3.44 | 163.0388, 135.0441, 145.0283 | 1-o-caffeoylquinic acid* |
| 19 | 12.78 | C_10_H_10_O_4_ | 195.0647 | 195.0652 | M+H | -2.37 | 195.0872, 163.0388, 113.9641, 131.9743, 135.0440 | Methyl caffeate* |
| 20 | 12.79 | C_21_H_20_O_11_ | 447.0933 | 447.0933 | M-H | 0.04 | 447.0939, 255.0297, 284.0328, 227.0344, 285.0396 | Astragalin* |
| 21 | 13.45 | C_34_H_30_O_15_ | 677.1515 | 677.0512 | M-H | 0.55 | 191.0547, 179.0334, 353.0881, 173.0439, 515.1198, 497.1092 | 3,4,5-tricaffeoylquinic acid* |
| 22 | 13.45 | C_30_H_46_O_16_S_2_ | 725.2163 | 725.2154 | M-H | 1.18 | 646.2624,645.2603, 96.9576, 725.2167 | Atractyloside |
| 23 | 13.60 | C_25_H_24_O_12_ | 515.1186 | 515.1195 | M-H | 0.37 | 191.0548, 353.0886, 173.0439, 135.0430, 179.0333, 93.0320 | 4,5-dicaffeoylquinic acid* |
| 24 | 14.21 | C_25_H_24_O_12_ | 515.1194 | 515.1195 | M-H | -0.57 | 191.0548, 353.0880, 179.0334, 135.0430, 335.0788, 93.0320 | 3,4-dicaffeoylquinic acid* |
| 25 | 15.10 | C_15_H_20_O_3_ | 249.1477 | 249.1485 | M+H | -3.23 | 249.1482, 145.1010, 105.0706, 231.1379, 119.0860 | Tomentosin* |
| 26 | 16.39 | C_30_H_46_O_13_S | 645.2589 | 645.2586 | M-H | 0.50 | 645.2602, 96.9576, 101.1584, 647.2619,646.2634 | 4'-desulphate -atractyloside |
| 27 | 22.57 | C_18_H_30_O_2_ | 279.2308 | 279.2319 | M+H | 1.04 | 95.0864, 81.0710, 279.2316, 261.2206, 243.2106 | Linolenic acid* |

Note: * Referring to identification by comparison with a reference standard.

## Table. S2 Potential biomarkers for serum metabolomics screening

| **Ion mode** | **Retention time (min)** | **Compound name** | **Chemical formula** | ***m*/*z*** | **Error (ppm)** | **AAM vs SHA** | **AAM vs XFL** | **Metabolic pathway** |
| --- | --- | --- | --- | --- | --- | --- | --- | --- |
| ESI+ | 9.20 | Pyridoxamine | C_8_H_12_N_2_O_2_ | 359.17 | 3.2 | ↓^**^ | ↑^##^ | Vitamin B_6_ metabolism |
|  | 10.11 | Estrone sulfate | C_18_H_22_O_5_S | 701.24 | -0.9 | ↑^**^ | ↓^##^ | Steroid hormone biosynthesis |
|  | 10.30 | Inosinic acid | C_10_H_13_N_4_O_8_P | 735.06 | -0.5 | ↓^**^ | ↑^##^ | Purine metabolism |
|  | 3.38 | LysoPC(16:0) | C_24_H_50_NO_7_P | 496.34 | 3.0 | ↑^**^ | ↓^##^ | Glycerophospholipid metabolism |
|  | 8.30 | Lactosylceramide (d18:1/12:0) | C_42_H_79_NO_13_ | 828.55 | 4.4 | ↑^**^ | ↓^##^ | Glycerophospholipid metabolism |
|  | 8.21 | Sphingomyelin | C_41_H_83_N_2_O_6_P | 731.61 | 4.7 | ↓^**^ | ↑^##^ | Sphingolipid metabolism |
|  | 8.48 | Anthranilic acid | C_7_H_7_NO_2_ | 297.08 | 2.4 | ↑^**^ | ↓^##^ | Tryptophan metabolism |
|  | 10.98 | Farnesyl pyrophosphate | C_15_H_28_O_7_P_2_ | 787.25 | 0.7 | ↑^**^ | ↓^##^ | Steroid biosynthesis |
| ESI- | 8.32 | Thyroxine | C_15_H_11_I_4_NO_4_ | 775.68 | -2.5 | ↓^**^ | ↑^##^ | Tyrosine metabolism |
|  | 8.41 | Thyroxine | C_15_H_11_I_4_NO_4_ | 775.68 | -2.5 | ↓^**^ | ↑^##^ | Tyrosine metabolism |
|  | 0.86 | Normetanephrine | C_9_H_13_NO_3_ | 365.17 | 3.1 | ↓^**^ | ↑^##^ | Tyrosine metabolism |
|  | 8.99 | Phosphorylcholine | C_5_H_15_NO_4_P | 165.06 | 5.0 | ↑^**^ | ↓^##^ | Glycerophospholipid metabolism |
|  | 5.70 | Lactosylceramide (d18:1/25:0) | C_55_H_105_NO_13_ | 986.75 | 2.3 | ↑^**^ | ↓^##^ | Sphingolipid metabolism |
|  | 1.31 | α-Lipoic acid | C_8_H_14_O_2_S_2_ | 187.02 | -2.8 | ↓^**^ | ↑^##^ | Lipoic acid metabolism |
|  | 1.04 | Citric acid | C_6_H_8_O_7_ | 173.01 | -4.7 | ↓^**^ | ↑^##^ | TCA cycle |
|  | 1.76 | Chenodeoxycholic acid | C_24_H_40_O_4_ | 391.28 | -1.3 | ↓^**^ | ↑^##^ | Primary bile acid biosynthesis |
|  | 3.75 | Deoxycorticosterone | C_21_H_30_O_3_ | 311.20 | -3.9 | ↓^**^ | ↑^##^ | Steroid hormone biosynthesis |

Note: Compared with SHA group, ^**^*p*<0.01, compared with AAM group, ^##^*p*<0.01.
